# Levels of Mineral Elements in Different Organs of Dogs from the Ionian-Etnean Volcanic Area

**DOI:** 10.3390/ani15111545

**Published:** 2025-05-25

**Authors:** Fabio Bruno, Anthea Miller, Giuseppe Bruschetta, Vincenzo Nava, Claudia Rifici, Sebastiano Zappalà, Patrizia Licata

**Affiliations:** 1 Department of Veterinary Sciences, University of Messina, 98168 Messina, Italygiuseppe.bruschetta@unime.it (G.B.);; 2 Department of Biomedical and Dental Sciences and of Morphological and Functional Imagines (BIOMORF), University of Messina, 98168 Messina, Italy

**Keywords:** dog, levels, mineral elements, statistical analysis, sentinel animals, volcanic area

## Abstract

Mineral elements can either be pollutants or essential dietary components. Monitoring their levels in the environment and living organisms is crucial because excessive amounts can become toxic. Dogs, due to their proximity to humans, shared habitats, and similar organ structures, can be effective indicators of environmental pollution by toxic elements. This study aimed to assess the levels of 11 mineral elements in 80 dog carcasses (49 males and 31 females), aged between 2 and 16 years, from the Ionian-Etnean volcanic region of the province of Catania, where the dogs had died under unknown circumstances. A direct mercury analyzer (DMA-80) was used to measure Hg, and an inductively coupled plasma mass spectrometer (ICP-MS) was used for the other elements. A one-way ANOVA, followed by Bonferroni’s multiple comparison for post hoc analysis, was conducted to evaluate significant differences between the organ samples and different minerals and between the weight and metal levels. The statistical significance was set at *p* < 0.05. The study indicates that high concentrations of metals like cadmium, mercury, lead, and chromium are present in the liver, kidneys, and other organs. These elevated concentrations suggest that the local volcanic emissions contribute to soil, water, and atmospheric contamination. The data showed differences in the metal concentrations between the sexes, which could be attributed to biological and environmental factors.

## Simple Summary

The presence of mineral elements in the environment can be attributed to both anthropogenic and non-anthropogenic sources of pollution. The purpose of this study was to assess the concentrations of mercury, cadmium, lead, cobalt, copper, bismuth, chromium, vanadium, nickel, zinc, and iron in different organs of dogs from the Ionian-Etnean volcanic region of the Catania province. Studies have shown that people living on Mount Etna’s slopes are chronically exposed to elevated levels of metal mixtures, especially in the Ionian region, where higher concentrations of heavy metals have been found in the water. The study aimed to utilize dogs as sentinel animals of environmental pollution, with an emphasis on factors that contribute to the mortality of animals living in rural areas. Statistical analysis was performed in organ samples of dogs. Results showed statistically significant differences between organ samples, different minerals, and between the weight and metal levels.

## 1. Introduction

Mineral elements, such as iron (Fe), copper (Cu), manganese (Mn), zinc (Zn), selenium (Se), and iodine (I), are naturally occurring elements that play essential roles in various metabolic processes and biological signaling pathways. However, these elements can become toxic if ingested in excessive doses. Conversely, other metals, including cadmium (Cd), lead (Pb), mercury (Hg), arsenic (As), and nickel (Ni), are non-essential, lack biological functions, and exhibit toxicity at very low concentrations. These metals are classified as toxic heavy metals [1].

Environmental pollution by heavy metals, arising both from the natural abundance of metals in the Earth’s crust and from anthropogenic activities, holds significant toxicological importance due to the broad spectrum of pathological effects on humans and animals. Chronic exposure to heavy metals is commonly associated with conditions such as carcinogenicity, teratogenicity, immunosuppression, and reproductive impairments [2,3]. The presence of metals in the environment can be attributed to both anthropogenic and non-anthropogenic sources of pollution [4]. Non-anthropogenic heavy metal pollution, as a result of gas, ash, and lava emissions, has been observed in various volcanic regions [5]. Metals and their traces can pose severe risks to the environment and public health, particularly in urban areas located near active volcanoes. These metals accumulate in soils, plants, and groundwater, which may also be used for drinking purposes. Mount Etna, the largest active volcano in Europe, has been documented to emit large quantities of gases and ashes, along with heavy metals [6,7]. These emissions contribute to soil, water, and atmospheric contamination [8,9,10], and the metals can enter the food chain, as evidenced by the presence of elevated levels of metals in the urine and scalp hair of individuals living in areas adjacent to the volcano, compared with those in non-volcanic regions [11,12].

Moreover, studies have shown that residents living on the slopes of Mount Etna are chronically exposed to increased levels of a mixture of metals, particularly in the Ionian region, where significantly higher concentrations of several heavy metals have been detected in the groundwater [8,13]. The correlation between volcanic-origin chemical elements and the health of populations residing on the slopes of the volcano has been investigated. Results regarding metal deposits in the soil and groundwater contamination suggest that volcanic emissions from Etna could significantly impact human health, contributing to an increase in the incidence of neurodegenerative diseases, including multiple sclerosis and amyotrophic lateral sclerosis, as well as several types of cancer such as thyroid cancer [14,15,16,17]. While domestic animals are inevitably exposed to the same environmental pollutants, making them important sentinels for toxicological risk assessment and public health as well as valuable models for translational medicine [18,19], no studies have yet been conducted on the potential correlation between non-anthropogenic pollution in volcanic areas and heavy metal-related pathologies in domestic animals.

The aim of this study was to evaluate the levels of mercury (Hg), cadmium (Cd), lead (Pb), cobalt (Co), copper (Cu), bismuth (Bi), chromium (Cr), nickel (Ni), zinc (Zn), and iron (Fe) in dog cadavers from the Ionian-Etnean volcanic region of the province of Catania. Water samples were also analyzed for the determination of toxic elements (As, Cd, Cr, Hg, Ni, Pb). Furthermore, the study aimed to use dogs as surrogate indicators of metal exposure.

## 2. Materials and Methods

### 2.1. Animals and Sampling Area

The Department of Veterinary Sciences at the University of Messina conducted necropsies on 80 dog cadavers (49 males and 31 females) with ages ranging from 2 to 16 years, from May 2023 to July 2024. The cadavers came from a shelter located in the province of Catania, where the dogs died (Table 1). The shelter is situated in the countryside on the northern outskirts of Acireale (CT) and is dedicated to housing stray or abandoned dogs. The facility was designed to accommodate approximately 150 dogs, in accordance with regulations governing such establishments. However, the shelter contains 70 tiled kennels of varying sizes, including both enclosed and open areas, which allow the dogs to experience different levels of exposure to the elements. Upon arrival at the shelter, each dog undergoes a comprehensive veterinary examination to assess its general health and identify any immediate health issues. Additionally, as part of routine care, the dogs are treated for internal and external parasites upon admission, with follow-up treatments provided as necessary. Vaccination programs are maintained based on the dogs’ age and health status, ensuring that they receive the required immunizations throughout the year. Feeding is provided once daily, typically at midday, with each dog receiving approximately 300 g of commercial dry food along with approximately 3 L of fresh tap water. This feeding regimen aims to meet their basic nutritional requirements, although variations may occur depending on individual health conditions, specific veterinary recommendations, or seasonal adjustments to food and water intake.

### 2.2. Sample Collection

A total of eighty samples of liver, kidney, heart, muscle (longissimus dorsi), and lung were taken from the dogs, regardless of the cause of death or any pathological findings observed in the tissues. This study was carried out to assess the presence of metals in surface and groundwater within a representative area on the northeastern slope of Mount Etna. A total of fifteen samples of groundwater were taken from three selected points, as the source of supply for the municipal water distribution system. Water samples were collected using high-density polyethylene (HDPE) bottles previously cleaned with 10% nitric acid and rinsed with ultrapure deionized water. Powder-free nitrile gloves were used during all sampling activities to minimize the risk of contamination. Samples of the organs were collected in plastic containers and stored at −20 °C until analysis. These organs were subsequently analyzed for the determination of mineral elements including mercury (Hg), cadmium (Cd), lead (Pb), cobalt (Co), copper (Cu), bismuth (Bi), chromium (Cr), vanadium (V), nickel (Ni), zinc (Zn), and iron (Fe). The water samples were diluted and acidified using 2% nitric acid. The blank and certified reference material (CRM) samples underwent the same treatment and were processed alongside each batch of digested samples. All samples were filtered through a 0.50 μm filter to eliminate larger particles. Each determination was performed in triplicate.

### 2.3. Mineralization ICP-MS and DMA-80 Analysis

Samples were prepared according to Nava et al. (2023) with some modifications (i.e., by mineralization (or acid digestion) using a “Milestone ETHOS 1” microwave mineralizer, Milestone Technologies Inc., Fremont, CA, USA) [18]. For the mineralization of canine biological samples, approximately 0.5 g was weighed and then digested by adding 8 mL of 65% nitric acid (HNO_3_) and 2 mL of 30% hydrogen peroxide (H_2_O_2_). The analytical blank and certified reference materials (ERMBB184-bovine muscle) were prepared in the same way. Digestion was performed in two stages: the first involved a linear increase in temperature to 180 °C for 15 min at a maximum power of 1000 W, while the second involved holding the temperature constant at 180 °C for 15 min at the same power. All samples were filtered using a syringe and 0.45 μm filters. All samples were analyzed by inductively coupled plasma-mass spectrometry using a Thermo Scientific iCAP Q-ICP-MS (Thermo Fisher Scientific, Waltham, MA, USA), with operating conditions similar to those reported by Bruno et al. (2024a) [20] and Bruno et al. (2024b) [21]. A Direct Mercury Analyzer (DMA-80, Milestone) was used for Hg detection. The operative procedure followed those indicated by Messina et al. (2024) [22]. The sample (0.1 g for solid samples and 100 μL for liquid samples) was introduced into the furnace where it was dried (250 °C for 60 s) and then thermally decomposed in an oxygen stream (750 °C for 150 s). The quantitative determination of mercury was carried out by atomic absorption at 253.65 nm.

### 2.4. Calibration Procedure

The following standard solutions were used for ICP-MS analysis: Bi, Cd, Co, Cr, Cu, Fe, Ni, Pb, V, Zn (1000 mg/L in 2% nitric acid) from Fluka (Milan, Italy). Standards were used to construct the calibration curves. Specifically, seven-point calibration curves were obtained using standard solutions at the following concentrations: 0.5, 2.0, 1.0, 5.0, 10.0, 20.0, and 50.0 μg/L. Standard solutions of ^45^Sc, ^73^Ge, and ^115^In (1000 mg/L in 2% nitric acid, Fluka) were used as online internal standards (at the level of 1.5 mg/L) to correct for instrumental drift and matrix variations. An Re standard solution at 1000 mg L^−1^ in 2% nitric acid (Fluka) was used as a preparation standard at 0.5 mg/L to check sample digestion and correct for volumetric variations. A solution containing 1 μg/L of ^7^Li, ^59^Co, ^138^Ba, ^209^Bi, ^142^Ce, ^115^In, and ^238^U in 2% HNO_3_ + 0.5% HCl (Thermo Fisher Scientific, Waltham, MA, USA) was used for instrument calibration. The isotopes monitored were ^51^V, ^52^Cr, ^56^Fe, ^59^Co, ^60^Ni, ^63^Cu, ^66^Zn, ^114^Cd, ^208^Pb, and ^209^Bi. For mercury, calibration curves were established using a certified mercury standard at 1000 mg/L (CZECH Metrology Institute Analytika, Brno, Czech Republic). Standard solutions were then prepared in the range 0.3–100.0 μg/L. Three certified matrices were used to validate the method in terms of linearity (R^2^), sensitivity (LOD and LOQ), and accuracy (% recovery): ERMBB184-bovine muscle for organs (different fortification levels: 0.1, 0.2, and 0.5 mg/L for Pb, Ni, Cr, Cd, Bi, and Hg; and 0.5, 2.0, and 5.0 mg/L for Fe, Zn, Cu, and Co) (Table 2).

### 2.5. Statistical Analysis

G Power 3.1 software was used to determine the sample size for the “a priori” ANOVA test (fixed effect, omnibus, one-way), with an effect size (f) of 0.40, a significance level (α) of 0.05, a power (1-β) of 0.80, and five groups. Statistical analysis was carried out using GraphPad Prism 9.0 (GraphPad Software, Inc., Boston, MA, USA). Shapiro–Wilk normality was performed, and data were reported as the mean ± SD. Differences among samples were assessed by one-way ANOVA, followed by the Bonferroni test, and the correlation between the weight and mineral levels were evaluated by Pearson’s correlation. The statistical significance was set at *p* < 0.05.

## 3. Results

**Figure 1:**
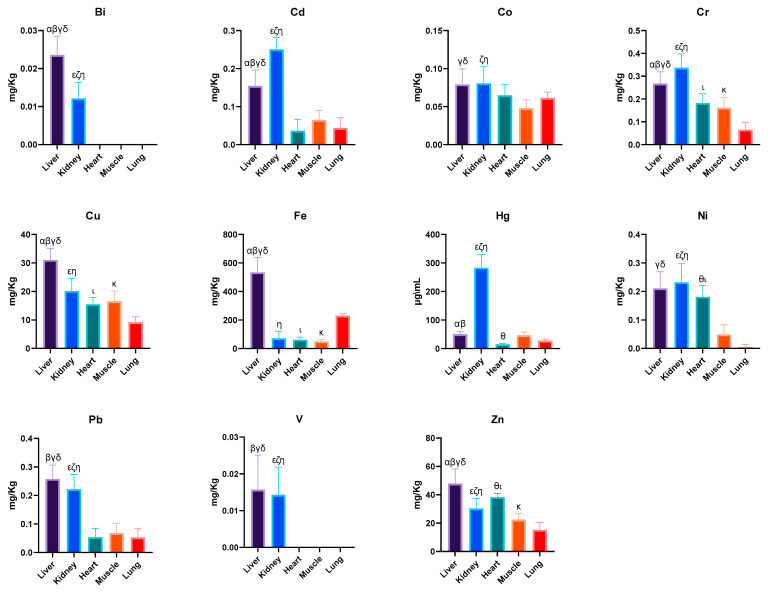
Concentrations of mineral elements in the dog samples. Differences between liver and kidney ^α^; differences between liver and heart ^β^; differences between liver and muscle ^γ^; differences between liver and lung ^δ^; differences between kidney and heart ^ε^; differences between kidney and muscle ^ζ^; differences between kidney and lung ^η^; differences between heart and muscle ^θ^; differences between heart and lung ^ι^; differences between muscle and lung ^κ^. Data are expressed as the mean ± standard deviation.

In our study, the levels of mineral elements were analyzed in the organ samples of dogs from the Ionian-Etnean volcanic area. The concentrations (mean ± SD) of the mineral elements reported in dogs are shown in Table 3 and Figure 1.

The concentration of Bi in the liver was significantly higher than in the kidneys (*p* < 0.0001). Levels of Bi were below the detection limit in the hearts, muscles, and lungs.

Cadmium concentration in the liver was significantly lower than in the kidneys (*p* < 0.0001). The heart contained lower concentrations of Cd than liver, followed by the muscles and lungs (*p* < 0.0001). The cobalt level found in the liver samples was slightly lower than the kidneys. The muscles and lungs had lower concentrations than the hearts. The liver showed a lower Cr concentration than the kidney samples (*p* < 0.0001). The heart contained lower Cr levels than the liver (*p* < 0.0001) and kidneys (*p* = 0.0026), followed by the muscles and lungs. The liver samples showed the highest level of Cu, followed by the kidneys (*p* < 0.0001). The heart contained a slightly lower concentration of Cu than the kidneys (*p* = 0.0066), while the muscles reported levels similar to the heart samples, and the lungs exhibited the lowest Cu concentrations (*p* < 0.0001). Regarding the liver samples, a high Fe concentration was reported that was significantly higher than the other organs (*p* < 0.0001), the kidneys contained a lower concentration than the lungs (*p* < 0.0001), while the heart showed values similar to the kidneys. The muscles had a lower concentration than the heart and kidneys. The lungs showed higher values than in the heart and muscles (*p* < 0.0001), but were still lower than the liver (*p* < 0.0001). The Hg concentration in the liver was lower than the kidneys (*p* < 0.0001), and the heart samples contained a low value of Hg, significantly lower than the kidneys (*p* < 0.0001) and liver (*p* = 0.0006). The muscle samples showed a higher Hg concentration than the lungs.

With regard to the concentration of Ni in the liver, it showed a high value compared with other organs, while the kidneys exhibited a higher concentration than the liver. The heart contained lower levels than the kidneys, muscle, and lungs (*p* < 0.0001).

Lead concentration in the liver samples exhibited high values compared with other organs, while the kidneys reported a lower concentration than the liver (*p* < 0.0001). The heart had a low value of Pb, followed by the muscles and lungs (*p* < 0.0001).

Kidneys samples showed a low concentration of V compared with the liver. A higher concentration of V was observed in the heart than in the kidneys (*p* < 0.0001). The muscles showed negligible levels, and the lungs reported V concentrations that were below the LOQ.

The liver had a higher Zn concentration compared with other organs (*p* < 0.0001), and the kidney samples contained lower levels than the heart (*p* < 0.0001). The muscles and lungs showed the lowest concentration of Zn (*p* = 0.0439).

Statistically significant correlations between body weight and the concentration of mineral elements were observed. In particular, a significant negative correlation was found between body weight and Bi concentration in the kidneys (r = −0.295, *p* = 0.008). For Cd, a positive correlation was observed in the heart (r = 0.237, *p* = 0.034). Cobalt concentration in the kidneys was positively correlated with body weight (r = 0.393, *p* = 0.0003), and chromium showed a significant positive correlation with body weight in the heart (r = 0.229, *p* = 0.041), while Cu exhibited a similar trend in the liver (r = 0.225, *p* = 0.045). Iron concentrations were positively correlated with body weight in multiple organs: kidneys (r = 0.309, *p* = 0.005), heart (r = 0.295, *p* = 0.008), and muscle tissue (r = 0.305, *p* = 0.006). Mercury levels also showed significant positive correlations in the liver (r = 0.331, *p* = 0.003) and heart (r = 0.339, *p* = 0.002). No statistically significant correlations were found for the Ni and V concentrations in relation to body weight. Lead values reported positive correlations with body weight in muscle (r = 0.305, *p* = 0.006) and lung tissue (r = 0.291, *p* = 0.009). Finally, a significant positive correlation was observed between body weight and Zn concentration in the kidneys (r = 0.301, *p* = 0.007).

Heavy metal concentrations in the water samples are shown in Table 4.

**Figure 2:**
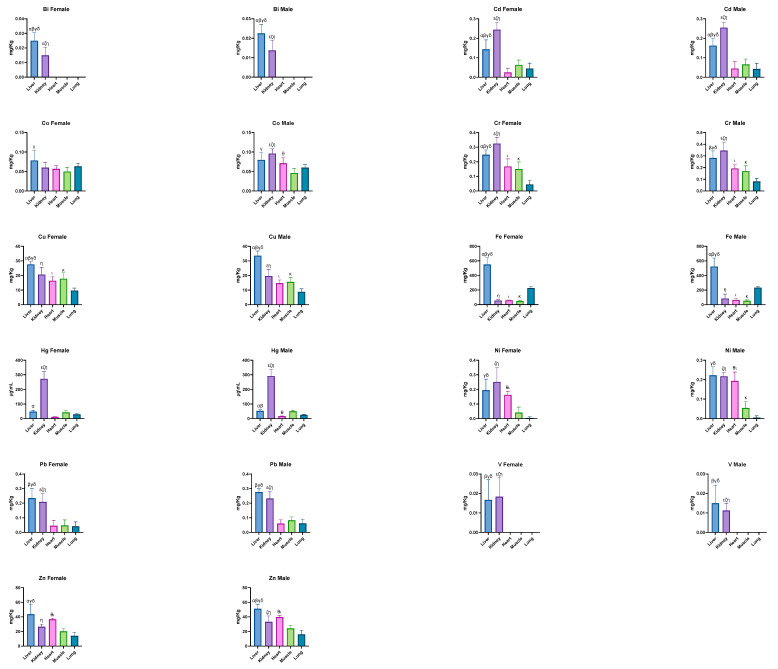
Mineral elements in female and male dogs. Differences between liver and kidney ^α^; differences between liver and heart ^β^; differences between liver and muscle ^γ^; differences between liver and lung ^δ^; differences between kidney and heart ^ε^; differences between kidney and muscle ^ζ^; differences between kidney and lung ^η^; differences between heart and muscle ^θ^; differences between heart and lung ^ι^; differences between muscle and lung ^κ^. Data are expressed as the mean ± standard deviation. Data are represented as the mean ± SD.

The concentrations (mean ± SD) of the mineral elements reported in the female and male dogs are shown in Figure 2.

The comparison of the mineral element concentrations in different organs in relation to sex showed that Bi had similar values in both sexes. In the liver, females exhibited higher concentrations of Bi compared with males (*p* < 0.0001). In both sexes, the Bi levels in the heart, muscles, and lungs were below the limit of quantification.

Cadmium showed variable concentrations in the dog organs, with some differences observed between sexes. In the liver, males exhibited slightly higher Cd levels than females. Cadmium concentrations were also slightly higher in males compared with females, with both sexes showing significantly higher levels in the kidneys than in others organs (*p* < 0.0001). In the heart, the Cd concentrations in male dogs were higher than in females. Similarly, muscle samples showed higher levels in males than in females. On the contrary, the Cd levels in the lungs were slightly higher in females than in males.

Regarding the Co concentrations, the females and males showed similar levels in the liver. In both sexes, the Co levels in the liver were significantly higher than in the muscle tissue (*p* < 0.0001), and in male dogs, the liver showed significantly higher Co concentrations compared with other organs (*p* < 0.0001). In the heart, the Co levels in males were higher than in the muscle tissue, with a statistically significant difference (*p* < 0.0001).

Chromium values in the liver were higher in males than in females, with both sexes showing significant differences in the liver compared with the heart, muscle, and lungs (*p* = 0.0108; *p* = 0.0008; *p* < 0.0001, respectively). Chromium concentrations were higher in the kidneys than in all other organs for both sexes, with statistically significant differences compared with the heart, muscle, and lungs (*p* < 0.0001). In the heart, Cr levels were higher in males than in females, and both sexes exhibited significantly higher values in the heart than in the lungs (*p* < 0.0001). Chromium values were higher in males compared with females, with significant differences in muscle tissue compared with the lungs for both sexes (*p* < 0.0001).

Copper concentrations in the liver were higher in males than in females, showing a significant difference in this organ compared with the kidneys, heart, muscle, and lungs (*p* = 0.0124; *p* < 0.0001; *p* = 0.0003; *p* < 0.0001, respectively). In the kidneys, the Cu levels were slightly higher in the females than in males, and both sexes showed significantly higher Cu concentrations in the kidneys compared with the lungs (*p* < 0.0001). The values of Cu in male kidneys showed a significant difference than the heart (*p* = 0.0316). In the heart, the Cu levels were higher in the females than in males, and both showed significantly higher concentrations in the heart compared with the lungs (*p* = 0.0051 for males and *p* = 0.0154 for females, respectively). In the muscle tissue, the Cu levels were higher in the females than in males, with significantly higher concentrations than those found in the lungs for both sexes (*p* = 0.0024 for females and *p* = 0.0010 for males).

Iron concentrations in the liver were higher in the females than in males, and both sexes showed a significant difference in the liver compared with the kidneys, heart, muscle, and lungs (*p* < 0.0001). In the kidneys, the Fe levels were higher in the males than in females, with significant differences observed in both sexes compared with the lungs (*p* < 0.0001). In the heart, the Fe values were higher in the males than in females, and both sexes reported significantly higher levels in the heart compared with the lungs (*p* < 0.0001 for females and *p* = 0.0002 for males). In the muscle tissue, the Fe concentrations were higher in males than in females, with significant differences observed for both sexes compared with the lungs (*p* < 0.0001).

Mercury concentrations in the liver of males were higher than in females, with significant differences observed in the liver of both sexes compared with the kidneys (*p* < 0.0001). The Hg value in the liver samples of the males showed significant differences in the heart (*p* = 0.0190). In the kidneys, the Hg levels were higher in the males than in females, with significant differences reported in both sexes compared with the heart, muscle, and lungs (*p* < 0.0001). In heart samples of the males, the Hg levels showed significant differences toward muscle (*p* = 0.0371).

Nickel concentrations in the liver were slightly higher in the males than in females, with significant differences observed in both sexes compared with muscle and lungs (*p* < 0.0001 for males; *p* = 0.0014 and *p* < 0.0001, respectively, for females). In the kidneys, the Ni levels were higher in the females than in males, with significant differences for both sexes in relation to muscle and lung tissue (*p* < 0.0001). Nickel concentrations in the heart were higher in the males than in females. Males and females showed significant differences in relation to muscle and lung tissue (*p* = 0.0149 and *p* = 0.0009, respectively, for females and *p* < 0.0001 for males). In the muscle samples, the Ni levels in males were lower than in the other organs, with significant differences compared with the lungs.

In the liver samples, the lead concentrations were higher in the males than in females. Both sexes showed higher levels in the liver compared with the heart, muscle, and lungs (*p* < 0.0001). Similarly, in the kidneys, the Pb concentrations were slightly higher in the males than in females, and both sexes reported significant higher levels compared with the heart, muscle, and lungs (*p* < 0.0001).

Female dogs showed higher levels of V in the liver than in the males. Both sexes reported significantly higher levels related to the heart, muscle, and lungs (*p* < 0.0001). In the kidneys, the V levels were higher in the females than in males, with significant differences observed in both sexes than the heart, muscle, and lungs (*p* < 0.0001).

As for the zinc levels in the liver samples of females, they showed significant differences compared with the kidney, muscle, and lung (*p* = 0.0021; *p* < 0.0001; *p* < 0.0001, respectively). In males, the zinc levels reported significant differences in the kidneys, heart, muscle tissue, and lungs (*p* < 0.0001; *p* = 0.0029; *p* < 0.0001; *p* < 0.0001, respectively). The zinc concentrations in the kidneys of the females and males had significant differences toward lungs in females (*p* = 0.0473) and significant differences toward muscle and lungs for males (*p* = 0.0327; *p* < 0.0001). In the hearts, the zinc concentrations in the females and males showed significant differences toward the muscle and lung for both sexes (*p* = 0.0031; *p* < 0.0001, respectively, for females and *p* < 0.0001 for males).

## 4. Discussion

Within dogs, the Cd concentrations were significantly higher in the kidneys compared with the liver (0.251 vs. 0.154 mg/kg). This pattern is consistent with previous studies conducted on dogs in northwestern Spain, where the kidneys showed mean Cd levels of 0.166 mg/kg, exceeding those found in the liver (0.056 mg/kg) [23]. Other authors described cadmium levels in the liver of 0.098 mg/kg, which were lower than the values found in our samples. Regarding the cadmium concentrations found in the kidney samples, these levels were similar to those reported by Esposito et al. (2019) [24]. Similarly, research carried out in Naples reported average renal concentrations of 0.259 mg/kg and hepatic concentrations of 0.093 mg/kg [25]. These findings confirm the tendency of Cd to preferentially accumulate in the kidneys.

The levels of Co in the liver and kidney samples found in this study were higher than those described by other authors, who reported values of 16.3 and 21.0 μg/kg in the liver and kidney, respectively [23]. Different studies assessing the accumulation of Co in different tissue remains low. It has been proposed that Co may promote tumorigenesis by simulating hypoxic conditions, thereby affecting processes such as angiogenesis and apoptosis, and by interfering with intracellular signaling pathways including the activation of estrogen receptor alpha (ERα) [26].

The levels of Cr contained in the liver and kidney samples were higher than those reported by Löpez-Alonso et al. (2007), who showed concentrations of 57.6 and 43.9 μg/kg, respectively [23]. Hexavalent chromium (Cr VI) has been classified as a Group I human carcinogen by the International Agency for Research on Cancer (IARC) [27]. Several studies have indicated that metals can accumulate in tissue and compromise genomic stability by inducing DNA damage, promoting gene mutations, impairing antioxidant defense mechanisms, and altering epigenetic regulation [28]. Moreover, certain metals are capable of binding to and activating estrogen receptors (ER), thereby stimulating cell proliferation and functioning as metalloestrogens [29].

The liver exhibited the highest Cu concentrations (31.04 mg/kg), followed by the kidneys (20.11 mg/kg). These values are in line with the physiological hepatic range reported for dogs (30–100 mg/kg) [30]. However, hepatic concentrations exceeding 100 mg/kg have been associated with chronic liver disease [31]. Our findings indicate that the Cu levels were within the physiological limits, although continued monitoring is recommended.

The liver samples showed the highest concentrations of Fe (534.4 mg/kg), consistent with its role as the primary iron storage organ. Previous studies have reported mean hepatic values of 394 mg/kg, suggesting that our data reflect a slight variation, potentially due to environmental factors [23]. On the contrary, Löpez-Alonso et al. (2007) reported iron levels in kidney samples that were lower than those shown in our samples [23].

Mercury concentrations in the kidney samples (283 μg/kg) determined in this study were significantly higher than the hepatic levels (50.59 μg/kg). Previous studies reported mean renal values of 51.9 μg/kg and hepatic concentrations of 32.7 μg/kg, while research conducted in Naples indicated mean hepatic levels of 0.054 mg/kg [23,25]. Our data suggest a more pronounced renal accumulation, potentially influenced by local environmental factors, as also demonstrated by high Hg concentrations found in the water samples analyzed.

The concentrations of Ni were higher in the kidneys (0.232 mg/kg) compared with the liver (0.211 mg/kg). These values exceeded those reported in studies on dogs in Spain (kidneys: 26.8 μg/kg; liver: 23.8 μg/kg), suggesting a potentially higher environmental exposure in our study area [23]. From a toxicological perspective, chronic Ni exposure is of concern due to its nephrotoxic potential, its capacity to generate reactive oxygen species (ROS), and its ability to disrupt cellular homeostasis. Prolonged Ni accumulation in renal tissue may lead to oxidative stress, mitochondrial dysfunction, and histopathological damage, raising concerns about long-term renal impairment and systemic toxicity [32].

High Pb concentrations were observed in the liver samples (0.258 mg/kg) compared with other organs. Studies on stray dogs in Naples reported hepatic concentrations as high as 3.45 mg/kg, while research involving both domestic and stray dogs indicated average liver levels of 0.321 mg/kg [24,25]. The lead concentrations found in the liver samples were similar to those shown by Esposito et al. (2019) [24], but higher than the data found by Löpez-Alonso et al. (2007) [23]. Regarding the values obtained for the kidney, they were higher than those reported in the literature, suggesting a possible environmental contamination [23,24].

The vanadium concentration contained in the liver samples was 0.012 mg/kg, while the kidneys exhibited slightly lower levels than the liver (0.010 mg/kg). Although data on V in dogs are limited in the literature to date, studies on human tissues have reported mean renal concentrations of 1.91 mg/kg [33]. Moreover, Defourny et al. (2024) reported a value of 35.14 μg/kg in the breast tissues of healthy dogs, in contrast with values of 13.04 μg/kg reported in canine mammary tumors [34]. Our results suggest the presence of V in canine tissues, but further studies are needed to understand its impact. Some authors have highlighted the antineoplastic activity of V, as demonstrated by both in vitro and in vivo studies. In particular, V induced dose-dependent apoptosis in MCF-7 breast cancer cells [35]. Furthermore, the chemopreventive properties of V have also been confirmed in several experimental studies using chemically induced mammary cancer models in rats [36].

The liver samples contained the highest concentration of Zn (47.87 mg/kg), followed by the kidneys (30.29 mg/kg). These values are consistent with the role of liver as the primary site of Zn accumulation. Some authors showed that in healthy dogs, hepatic zinc concentrations averaged approximately 140 ± 25 mg/kg on a dry weight basis, with reported fresh weight values ranging from 10.150 to 54.707 mg/kg [37,38,39]. In comparison, the zinc levels in the kidney cortex and medulla ranged from 4.770 to 40.808 mg/kg and 2.805 to 18.828 mg/kg fresh weight, respectively [37,38,39]. The kidney concentrations observed in our study were higher than those previously reported by Pereira et al. (2021) [39]. However, it is known that hepatic disorders can affect the Zn levels, highlighting the need for monitoring in cases of liver abnormalities.

There are no studies reporting the bismuth concentrations in dog organs.

Regarding the determination of heavy metals in the water samples analyzed, the concentration of arsenic was below the detection limit (<LOD). The cadmium concentration was 0.66 ± 0.02 μg/L, which was above the environmental quality standard limit of 0.1 μg/L [40]. This suggests that the cadmium level exceeds the acceptable threshold for water quality. The concentration of total chromium was 2.30 ± 0.22 μg/L, which was also above the environmental quality standard of 0.2 μg/L [40]. This indicates a concern, as the chromium concentration exceeded the recommended limit. The hexavalent chromium was below the detection limit (<LOD). The mercury level was 18.02 ± 1.14 μg/L, which was higher than the environmental quality standard of 0.05 μg/L [40]. The nickel concentration was 0.92 ± 0.12 μg/L, which was below the environmental quality standard of 0.5 μg/L [40]. This indicates that the nickel level in the water samples was within the acceptable limits for water quality [40]. The lead was below the detection limit (<LOD).

At present, there are no established legal limits or regulatory standards for the concentration of heavy metals in the organs and tissues of dogs, despite the potential of these animals to serve as valuable sentinel species for monitoring environmental exposure. While dogs are increasingly considered as useful models for evaluating human health risks due to their proximity to human environments and shared exposure pathways, the absence of specific legal frameworks addressing the permissible levels of metals in canine tissues limits the ability to directly correlate animal data with human exposure thresholds. This gap highlights the need for further research and regulatory consideration to optimize the use of dogs as indicators for assessing environmental health risks and their implications for human populations.

A limitation of this study was the absence of long-term environmental monitoring data, which prevented a comprehensive assessment of chronic exposure to heavy metals in the volcanic area. Moreover, while the study suggests a possible association between heavy metals and the onset of metal-related diseases (including neurodegenerative disorders and cancers until dead), no direct clinical or pathological evaluations were conducted to confirm these links in the studied population. This is the first study of its kind conducted on dogs from the Etna region, and its findings should be interpreted as exploratory.

## 5. Conclusions

This study provides a comprehensive assessment of mineral elements in the heart, kidney, liver, lungs, and muscle tissues of dogs from the Etna region, a volcanic area potentially subject to environmental contamination. Our results revealed organ-specific accumulation patterns consistent with the previous literature, notably the preferential retention of cadmium and mercury in the kidneys, and copper, iron, and zinc in the liver. The elevated concentrations of certain metals (such as cadmium, chromium, and mercury) compared with published data from other regions suggest a possible influence of local environmental factors. Notably, the cadmium and chromium levels in regional water samples exceeded the established environmental quality standards, supporting the hypothesis of environmental exposure as a contributing factor.

The tissue concentrations of cobalt, nickel, and lead were also found to exceed the values reported in previous studies, raising toxicological concerns, especially in relation to renal function and potential carcinogenic mechanisms. Although vanadium was detected in both the liver and kidney tissues, its toxicological significance in canine populations remains unclear, warranting further investigation. Importantly, the copper and zinc levels were within the physiological ranges, although variations may reflect underlying metabolic or hepatic alterations.

Despite the absence of established reference thresholds for heavy metals in canine tissues, our findings underscore the potential of dogs as sentinel species for monitoring environmental pollutants. However, the lack of long-term exposure data and clinical–pathological correlation limits the ability to draw definitive conclusions regarding health outcomes. Future studies incorporating broader environmental sampling, clinical assessments, and histopathological analysis are necessary to better understand the health implications of chronic metal exposure in both animals and potentially exposed human populations.

## Figures and Tables

**Table 1:** Data of the age, sex, gender, and number of dogs.

Breed	Age	Sex	Total Animals	Weight (kg) *
Mixed-breed	7	m	6	22–25
Mixed-breed	8	f	7	13–18
Mixed-breed	10	f	5	20–25
Czechoslovakian Shepherd	10	m	8	26–39
Mixed-breed	8	f	6	15–20
Rottweiler	2	f	4	35–40
Golden Retriever	6	f	3	25–30
Mixed-breed	8	m	7	24–34
Mixed-breed	12	f	6	7–18
German Shepherd	12	m	4	30–35
Pitt Bull	5	m	5	16–30
Mixed-breed	15	m	8	23–27
Chihuahua	16	m	6	1.2–3
Mixed-breed	6	m	5	3–8

* Data are expressed as range.

**Table 2:** Analytical validation of the ICP-MS and DMA-80 methods.

Element	LOD (mg/L)	LOQ (mg/L)	R^2^	ERMBB184 Bovine Muscle (%)
Bi	0.0015	0.005	0.9995	96.50 ± 0.75 **
Cd	0.001	0.003	0.9999	102.70 ± 1.43
Co	0.002	0.007	0.9996	97.00 ± 0.80 **
Cr	0.0015	0.005	0.9996	95.55 ± 0.46 **
Cu	0.003	0.01	0.9995	98.75 ± 1.48
Fe	0.003	0.01	0.9995	97.25 ± 0.78
Hg *	0.300 *	1.000 *	0.9997	98.65 ± 0.55
Ni	0.0018	0.006	0.9998	97.15 ± 0.40 **
Pb	0.001	0.003	0.9999	101.50 ± 1.70 **
V	0.003	0.01	0.9995	96.00 ± 0.45 **
Zn	0.03	0.10	0.9997	98.50 ± 0.86

* LOD and LOQ reported in μg/L. ** Analytes not included in the certified matrix.

**Table 3:** Mineral element concentrations and inter-organ comparisons in dogs.

Metals	Organs				
	Liver	Kidney	Heart	Muscle	Lung
**Bi** (mg/kg)	0.024 ± 0.005 ^α,β,γ,δ^	0.012 ± 0.004 ^ε,ζ,η^	<LOQ	<LOQ	<LOQ
**Cd** (mg/kg)	0.154 ± 0.042 ^α,β,γ,δ^	0.251 ± 0.031 ^ε,ζ,η^	0.036 ± 0.031	0.065 ± 0.025	0.044 ± 0.027
**Co** (mg/kg)	0.079 ± 0.021 ^γ,δ^	0.081 ± 0.022 ^ζ,η^	0.065 ± 0.014	0.048 ± 0.011	0.061 ± 0.008
**Cr** (mg/kg)	0.268 ± 0.052 ^α,β,γ,δ^	0.337 ± 0.061 ^ε,ζ,η^	0.182 ± 0.042 ^ι^	0.161 ± 0.046 ^κ^	0.066 ± 0.032
**Cu** (mg/kg)	31.040 ± 3.998 ^α,β,γ,δ^	20.110 ± 4.465 ^ε,η^	15.470 ± 2.495 ^ι^	16.560 ± 3.600 ^κ^	9.181 ± 1.932
**Fe** (mg/kg)	534.400 ± 104.000 ^α,β,γ,δ^	72.510 ± 47.550 ^η^	62.080 ± 17.500 ^ι^	50.790 ± 11.020 ^κ^	230.900 ± 14.340
**Hg** (μg/kg)	50.590 ± 9.491 ^α,β^	283.000 ± 46.780 ^ε,ζ,η^	14.940 ± 2.830 ^θ^	47.280 ± 10.420	27.400 ± 5.705
**Ni** (mg/kg)	0.211 ± 0.058 ^γ,δ^	0.232 ± 0.065 ^ε,ζ,η^	0.181 ± 0.040 ^θ,ι^	0.049 ± 0.034	0.005 ± 0.009
**Pb** (mg/kg)	0.258 ± 0.049 ^β,γ,δ^	0.222 ± 0.051 ^ε,ζ,η^	0.054 ± 0.031	0.068 ± 0.033	0.053 ± 0.030
**V** (mg/kg)	0.012 ± 0.013 ^β,γ,δ^	0.010 ± 0.011 ^ε,ζ,η^	0.072 ± 0.197	0.001 ± 0.004	<LOQ
**Zn** (mg/kg)	47.870 ± 10.360 ^α,β,γ,δ^	30.290 ± 7.242 ^ε,ζ,η^	38.400 ± 2.522 ^θ,ι^	22.500 ± 4.342 ^κ^	15.250 ± 5.130

Differences between the liver and kidney ^α^; differences between liver and heart ^β^; differences between liver and muscle ^γ^; differences between liver and lung ^δ^; differences between kidney and heart ^ε^; differences between kidney and muscle ^ζ^; differences between kidney and lung ^η^; differences between heart and muscle ^θ^; differences between heart and lung ^ι^; differences between muscle and lung ^κ^. Data are expressed as the mean ± standard deviation.

**Table 4:** Heavy metals concentrations in the water samples.

	Heavy Metals (μg/L)					
	As	Cd	Cr	Hg	Ni	Pb
**Water**	<LOD	0.66 ± 0.02	2.30 ± 0.22	18.02 ± 1.14	0.92 ± 0.12	<LOD

Data are expressed as mean ± standard deviation.

## Data Availability

The data presented in this study are available on request from the corresponding author.
